# Feasibility and effectiveness assessment of a mobile application for subhealth management: Study protocol for a randomized controlled trial

**DOI:** 10.1097/MD.0000000000015704

**Published:** 2019-05-24

**Authors:** Seungwon Shin, Hyunjoo Oh, Minsu Kang, Minyoung Park, Byung-Hee Koh, Minwoo Hwang

**Affiliations:** a Clinical Trial Center, Korean Medicine Hospital, College of Korean Medicine, Kyung Hee University; b Medical and Oriental Comprehensive Healthcare Center, Kyung Hee University Hospital; c Department of Sasang Constitutional Medicine, Kyung Hee University Korean Medicine Hospital; d Department of Sasang Constitutional Medicine, Kyung Hee University Hospital at Gangdong; e Department of Sasang Constitutional Medicine, College of Korean Medicine, Kyung Hee University, Seoul, Republic of Korea.

**Keywords:** health service, mobile application, prevention and control, randomized controlled trial, traditional Korean medicine

## Abstract

**Introduction:**

*Mibyeongbogam* (MBBG) is a mobile application developed for subhealth status self-management in the Republic of Korea. It aims to assess a user's subhealth status, and then to recommend relevant traditional Korean medicine (TKM)-based health-promoting strategies. The purpose of this study is to evaluate the feasibility and effectiveness of MBBG's employment for the subhealth management of general healthy adults.

**Methods:**

This is a prospective, open-label, parallel group, randomized controlled trial that will seek to enroll 150 healthy adults, aged 30 to 49 years old, from 2 hospitals in the Republic of Korea. The eligible participants will then be randomly allocated to either the MBBG or control group, at a 1:1 allocation ratio. The MBBG group will use the application for 12 weeks, while the control group will undergo no intervention. The awareness of subhealth status will be primarily assessed. Health promoting behaviors, quality of life, TKM-based health questionnaires, and physical examination results will be assessed as secondary outcomes.

**Discussion:**

The primary endpoint will be tested with a 2-sample *t* test, or a Wilcoxon rank sum test. Any other continuous variables will be tested via an analysis of covariance, while categorical variables will be tested by a Chi-squared or Fisher exact test. Repeated measure analysis of variance will be performed to explore any in-group differences. The results will be addressed with a 95% confidence interval. We expect that MBBG will be the 1st TKM-based mobile application to be feasible for primary care in subhealth management.

**Trial registration:**

CRIS (Clinical Research Information Service), KCT0003488, February 11, 2019

## 1 Introduction

*Mibyeong* is a concept referring to subhealth status in traditional Korean medicine (TKM). This concept is usually used to mean a state in between health and disease. A person might complain of subjective symptoms or abnormal examination findings, but not be diagnosed with any disease, such as borderline hypertension or diabetes. This subhealth status does not require any specific treatment, but may carry with it a high risk for future disease development.^[1]^ Preventive medicine has been trying to find ways to manage this subhealth status.^[2]^ A survey involving 818 TKM practitioners showed that they often encountered patients with subhealth statuses, and these patients mostly complained of fatigue, pain, or digestion problems. However, it is also reported that only 23% people in the subhealth state go to see doctors to figure out what is happening with their bodies. About 73% to 80% of people who complained of either fatigue- or pain-related symptoms showed no improvements, after as long as 6 months.^[3]^ This is why TKM researchers have been trying to address the necessity of understanding,^[4]^ conceptualizing,^[1]^ and developing an instrument to identify subhealth states.^[5]^

*Mibyeongbogam* (MBBG) is a mobile application developed by the Korea Institute of Oriental Medicine, in Daejeon, within the Republic of Korea, to manage peoples’ subhealth statuses. It assesses an individual's state of health via a series of validated questionnaires, put together through several physical examination results, and then recommends how to, successfully, promote healthier lifestyles in TKM-based manners (meditation, exercising, herb teas, etc). Users can easily access the application via their smartphones, and can then be provided what they have to manage to improve their own general health, given information on their own health states.^[6]^

Clinical researchers have been trying to adopt mobile health technology aimed at positively changing health behaviors or to manage disease conditions. However, the evidence around it is not yet confirmed and the implementation of mobile applications for subhealth management is still rare.^[7]^ A previous study uncovered the possibility of a mobile application to help assess and manage youth mental health problems.^[8]^

The purpose of this study is to determine the effectiveness of MBBG employment for subhealth management by general healthy adults, and to assess its feasibility in implementing MBBG in practical ways.

## 2 Methods

This study follows the Standard Protocol Items: Recommendations for Interventional Trials (SPIRIT) 2013 statement.^[9]^ The Institutional Review Board of the Kyung Hee University Korean Medicine Hospital at Gangdong, in the Republic of Korea, reviewed and approved this study protocol, as well as its informed consent form (KHNMCOH 2018-07-002-002) on September 11, 2018 (protocol version 1.2). The protocol has been registered in the Clinical Research Information Service (CRIS, https://cris.nih.go.kr/cris/en/, KCT0003488) on February 11, 2019. Any protocol amendments will be publicly reflected via CRIS.

### 2.1 Objectives

The primary objective of this study is to compare the awareness around subhealth status between an MBBG group and a control group. Health promoting behaviors, physical markers, subjective health statuses (including a quality of life [QoL] scale) will be assessed and compared between the 2 groups. Based upon these results, the feasibility of the MBBG application in managing and preventing subhealth statuses within individuals will be assessed.

### 2.2 Study design and setting

**Figure 1:**
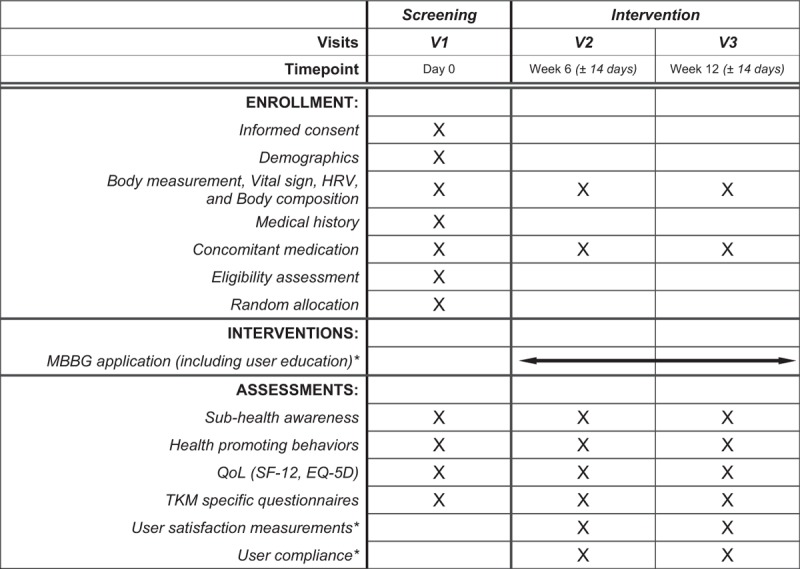
Study procedure. ∗Only for participants who are allocated to the intervention group. EQ-5D = EuroQoL 5-dimensional questionnaire, HRV = heart rate variability, MBBG = *Mibyeongbogam* (a mobile application), QoL = quality of life, SF-12 = short-form health survey 12-item, TKM = traditional Korean medicine.

This is a prospective, open-label, parallel group, randomized controlled trial. This study will seek to enroll 150 healthy adults, aged from 30 to 49 years, from 2 hospitals (Kyung Hee University Korean Medicine Hospital and Kyung Hee University Korean Medicine Hospital at Gangdong, Seoul) from the Republic of Korea. The study advertisements will be posted online and offline boards. The eligible participants will then be randomly allocated to either the MBBG or control group, at a 1:1 allocation ratio. The MBBG group will then use the application for a total of 12 weeks, while the control group will receive no intervention. All the outcomes will be assessed at the baseline, week 6, and, finally, at week 12. The study procedure is summarized in Figure 1.

### 2.3 Eligibility criteria

Healthy male and female adults, aged from 30 to 49 years old, who are capable of using mobile smartphones are eligible for this study. They should also be able to fill in self-report questionnaires, and evaluate physical biomarkers. If the participants do not own mobile smartphones with Android version 4.4 or higher or ones with iOS version 9 or higher, they will be excluded from the screening process. Any people assessed and found to have clinically significant medical conditions, from their medical history/concomitant medication reviews and physical examinations, will also be excluded from the study. If they are already using other mobile healthcare applications, they will also become ineligible. Participants who are involved in other trials within 1 month of this one, or are pregnant at baseline, will also be considered ineligible.

The TKM doctors will obtain written consent after all information around this study have been provided to participants. Personal information, including identification codes and names, will not be recorded in the case report forms nor will they be shared with others.

### 2.4 Randomization, allocation concealment, and blinding

An independent investigator will generate random sequence numbers using the R version 3.3.3 for Mac (The R Foundation) with the blockrand package, version 1.3. The random number and subsequently allocated group will then be written and delivered to each hospital via sealed envelopes. The investigators will open the envelopes in consecutive order, and assign an eligible participant to either the MBBG or the control group after a screening assessment is conducted in a 1:1 ratio. Since this is an open-label study, the participants and investigators will not be blinded. However, the outcome assessors will be blinded throughout the study to minimize possible bias.

### 2.5 MBBG application for the intervention group

The Korea Institute of Oriental Medicine, in Daejeon, within the Republic of Korea, has developed MBBG, a mobile application for subhealth management. MBBG aims at assessing a user's subhealth status, as well as their TKM-based health status, which leads it to recommend specific health promoting strategies, such as meditation, exercise, and herbal teas. Patients can check their subhealth status and TKM health information after submitting all of the necessary information, including questionnaire responses. The questionnaires are included within the application, so that the participants in the intervention group can successfully complete these via the application. The examination results (height, weight, vital signs, heart rate variability [HRV], etc) have to be input into the application manually.^[6]^

After their allocation to the intervention group, participants will be educated on how to use the application, verbally, and with a user manual during every visit. They are supposed to use MBBG at least once every day for a total of 12 weeks. The push notification function will be activated to motivate the participant to use the application throughout the study period. The history tracking and user ranking services are also available to help promote the use of MBBG. Participants are not allowed to use any other mobile applications for health management during study participation.

Compliance will be calculated based on how many times participants log onto the application during the 12-week study period. The user satisfaction level will be evaluated via 10 questions at visits 2 and 3, only for participants within the intervention group.

### 2.6 No intervention in the control group

Participants allocated to the control group go through the study without any intervention. They are told to maintain their usual lifestyles during the study period. They are also not supposed to use any other mobile applications for health management.

### 2.7 Outcomes

Data will be recorded in the form of case reports following its collection. Personal identification information will be coded. An independent monitor will regularly visit the study sites to gauge the eligibility confirmation, source data verification, protocol deviation check, and to evaluate overall user compliance.

The data sets used and/or analyzed after completing this study will be available from the corresponding author under all reasonable requests. The investigators will disseminate the study results via publication.

#### 2.7.1 Subhealth awareness

The primary outcome is the awareness of subhealth status. The participants will be given a questionnaire, consisting of 4 items: “Have you known or heard of subhealth statuses?” “Do you think that preventing diseases is as important as treating them?” “Do you think a professional medical service aimed at managing subhealth statuses is necessary?” “Are you willing to use a professional medical service to manage subhealth status, if available?” Each item is then scored from 1 (Not at all) to 7 (Absolutely), with the total score falling in the range of 4 to 28 points. The higher the score, the higher the awareness level. All participants are supposed to submit the subhealth awareness questionnaire during every visit (visits 1, 2, and 3) either via the mobile application or in a classic face-to-face fashion.

#### 2.7.2 Health promoting behaviors

The modified version of the health behavior scale, originally developed to evaluate health-promoting lifestyles,^[10]^ will be used for this study. This self-report scale consists of 25 items related to health responsibility, diet habits, exercise, stress management, and smoking habits. Each item has a 4-point response, from 1 (never) to 4 (always). The higher the score means that the individual more frequently performs healthy behaviors.^[11]^

The motivation scale for healthy behaviors will also be assessed using a self-efficacy questionnaire.^[10]^ The 5 questions therein will be on peoples’ abilities in avoiding greasy food, quitting smoking, exercising regularly, taking necessary medications, relieving mental stress, and obtaining health-related information, and the responses will be provided on a 5-point scale where 1 is Not confident at all to 5 is Absolutely confident.^[12]^

#### 2.7.3 QoL

The 12-item short-form health survey (SF-12), and EuroQoL Five Dimensions Questionnaire (EQ-5D) will both be used to assess the participants’ QoL. SF-12 consists of both physical and mental assessing components to evaluate general health status.^[13]^ EQ-5D is composed of the EQ-5D-5L and the EQ visual analog scale (VAS). EQ-5D-5L evaluates mobility, self-care, usual activities, pain/discomfort, and anxiety/depression, while EQ VAS rates a patient's health on a 10-cm vertical line.^[14]^

#### 2.7.4 TKM-specific questionnaires

Various TKM-based questionnaires are utilized to evaluate the subhealth status of each participant. The Mibyeong questionnaire is used to evaluate an individual's subhealth status. It assesses 4 physical symptoms (fatigue, pain, low sleep quality, and indigestion) and 3 mental distresses (anxiety, anger, and depression), of which, each item is scored from 1 to 7. An individual is considered as part of the healthy group if they achieve a total score of 26 points or less, while mild subhealth status are those with 27 to 47 points, and, finally, severe subhealth status are people scoring 48 points or more.^[5]^ The Sasang Constitutional type will be judged via a previously validated questionnaire, as well as a specialists’ clinical decision. The questionnaire will collect the usual states of a participant's appetite, digestion, urine and feces, cold/heat sensitivity, etc.^[15]^ Cold/heat sensitivities will also be assessed with a separate, validated questionnaire, as they are integral in figuring out how healthy a person is in terms of TKM.^[16]^

#### 2.7.5 Physical and social examination

Investigators will measure a participant's height, weight, vital signs (blood pressure, pulse rate, body temperature), pulse wave, HRV, and body composition (InBody) during every visit. Lifestyle activities, including alcohol-consumption, smoking, exercising, sleeping pattern, and eating habits, will also be investigated at the baseline.

### 2.8 Hypothesis and sample size calculation

The primary objective of this study is to compare the awareness levels of subhealth statuses between the MBBG and control groups. The null hypothesis is that there is no difference between the awareness score means between the groups. Since there have not been any previous studies implementing the MBBG application, we adopted another clinical trial to explore the mental health benefits of a mobile application. They addressed a 0.58 effect size with a 6-week test.^[8]^ This study set the intervention period as 12 weeks, and the age for the inclusion criteria is higher, and so we assumed the effect size to be, conservatively, 0.5. Therefore, the sample size has been calculated as 60 per group (2-sided, *α* = 0.05 and 1 − *β* = 0.8, independent *t* test) with G∗ power software version 3.1.3.^[17]^ With a 20% dropout rate expected, we will enroll 150 participants (75 in each group) to account for this.

### 2.9 Statistical analysis

The full analysis set is defined as a group including participants who are allocated to either the intervention or control group, use the MBBG application at least once, and assesses the primary outcome at least once. Per protocol set is defined as a group of the included participants who follow the trial procedure and demonstrate at least 70% compliance. The study results are analyzed primarily with the full analysis set, and subordinately with the per protocol set.

The primary variable in question is the difference in the subhealth status awareness levels between the baseline (visit 1) and the endpoint (visit 3). The mean difference will be tested using a 2-sample *t* test, or a Wilcoxon rank sum test. Any other continuous variables between the baseline and visits 2 or 3 will be tested independently, by an analysis of covariance with a covariate of the baseline values, while categorical variables will be tested by a Chi-squared or Fisher exact test. Repeated measure analysis of variance will be performed to explore any in-group differences between each of the variables throughout all visits. All of the results, analyzed with R software version 3.5.2 or later, will be addressed at a 95% confidence interval.

## 3 Discussion

This is a prospective, open-label, parallel group, randomized controlled trial aiming to evaluate the feasibility and effectiveness of a mobile application (MBBG) in managing subhealth statuses of healthy adults. One hundred fifty male or female subjects, without any specific diseases, will be allocated to either the MBBG or no-intervention group, with an allocation ratio of 1:1. Only the participants in the MBBG group will use the application for a total of 12 weeks. The awareness of subhealth status will be the variable primarily assessed, and changes to health promoting behaviors, QoL, TKM-based health questionnaires, and physical examination results will be also be statistically tested.

Mobile applications are a rising instrument aimed at helping people monitor their own health statuses, and in managing their own diseases, such as sleep disorders,^[18]^ diabetes,^[19]^ or even medication adherence.^[20]^ However, their effectiveness has not yet been confirmed.^[7]^ Also, no TKM-based application for health status monitoring, or subhealth management, exist. MBBG is the 1st mobile application, based on TKM theory as well as clinical research, that we expect will be feasible in the primary care for subhealth management.

## Author contributions

**Conceptualization:** Byung-Hee Koh, Minwoo Hwang.

**Data curation:** Seungwon Shin, Hyunjoo Oh.

**Funding acquisition:** Byung-Hee Koh, Minwoo Hwang.

**Investigation:** Hyunjoo Oh, Minsu Kang, Minyoung Park, Minwoo Hwang.

Hwang

**Methodology:** Seungwon Shin, Hyunjoo Oh.

**Visualization:** Seungwon Shin.

**Writing – original draft:** Seungwon Shin.

**Writing – review & editing:** Seungwon Shin, Byung-Hee Koh, Minwoo Hwang.

Minwoo Hwang orcid: 0000-0003-2928-9170.
